# Fracture Resistance of K3 Nickel-Titanium Files Made from Different Thermal Treatments

**DOI:** 10.1155/2016/6374721

**Published:** 2016-11-14

**Authors:** JinWook Choi, Soram Oh, Yu-Chan Kim, Kwang-Koo Jee, KeeYeon Kum, SeokWoo Chang

**Affiliations:** ^1^Department of Conservative Dentistry, School of Dentistry, Kyung Hee University, Seoul, Republic of Korea; ^2^Biomedical Research Institute, Korea Institute of Science and Technology, Seoul, Republic of Korea; ^3^Post-Silicon Semiconductor Institute, Korea Institute of Science and Technology, Seoul, Republic of Korea; ^4^Department of Conservative Dentistry, Dental Research Institute, Seoul National University Dental Hospital, Seoul National University School of Dentistry, Seoul, Republic of Korea

## Abstract

The purpose of this study was to compare fracture resistances of K3 nickel-titanium files made from different thermal treatments. K3 (SybronEndo, Orange, CA), K3XF (SybronEndo), and experimentally heat treated K3 (K3H) were used. For the cyclic fatigue test, the samples were rotated with up-and-down motion in the artificial canal with the curvature of 60 degrees until the fracture occurred. The number of cycles to fracture (NCF) was measured. For the torsional fracture test, the samples were tightly bound and rotated until the fracture occurred. Elastic modulus (EM), ultimate torsional strength (UTS), and angle of rotation to fracture (ARF) were measured. The results were statistically analyzed by one-way ANOVA. The NCF of K3H was higher than those of K3 and K3XF (*P* < 0.05). The EM of K3XF and K3H was lower than that of K3 (*P* < 0.05). There was no significant difference in UTS. The ARF of K3XF was higher than that of K3 (*P* < 0.05). K3XF and K3H showed more flexibility than K3. The maximum torsional angle of K3XF was higher than that of K3, but there was no significant difference on the UTS in all three groups.

## 1. Introduction

Nickel-titanium (NiTi) files offer increased flexibility and improved cutting efficiency [1]. Compared to conventional stainless steel files, there is a risk of unexpected fracture of NiTi files during clinical use. Unfortunately, these fractures of NiTi files have a potential effect on the outcome of treatment [2, 3]. Fracture of NiTi files can be categorized into two types: “torsional fracture” and “cyclic fatigue fracture” [4]. Torsional fracture occurs when a file tip is bound in the canal, but the motor continues to rotate. When the torque induced by the handpiece exceeds the ultimate torsional strength of a file, the file cannot withstand and finally fractures. Cyclic fatigue fracture occurs when a file rotates in curved canal receiving repeated compressive and tensile stresses [5].

Manufacturers have made efforts to increase the resistance to fracture, aiming at improving safety, through changes of design and manufacturing process [6, 7]. Recently, new thermal treatments during production of NiTi files were introduced and are being considered as new methods of improving fracture resistance of NiTi files [8].

NiTi alloy has three different microstructural phases that can change with temperature: “austenite,” “martensite,” and “R-phase.” They have several different properties. Austenite is present at a higher temperature, while martensite is present at a lower temperature. Austenite is quite strong and hard, while martensite is soft and ductile and can easily be deformed. R-phase is an intermediate phase present within very narrow temperature range between austenite and martensite. Mechanical properties of NiTi alloy are determined by the relative proportions and characteristics of the microstructural phases [8]. Conventional NiTi alloy is present in the austenite phase at room temperature. However, when the phase transition temperature changes by thermal treatment on NiTi alloy, martensite or R-phase or more than one phase can be present at room temperature.

Although a number of thermally treated NiTi files have been introduced recently, most of them have different geometric characteristics. There is a lack of knowledge to explain the effect of thermal treatment on the fracture resistance of files under same geometric condition.

In this study, we evaluated the fracture resistance of three kinds of K3 NiTi files (SybronEndo, Orange County, CA, USA), which are thermally treated in different ways. The purpose of this study was to compare fracture resistances of these NiTi files and to evaluate the effect of thermal treatment on fracture resistance of NiTi files.

## 2. Materials and Methods

### 2.1. Materials

Three kinds of NiTi files were selected: (i) K3 made of conventional NiTi alloy, (ii) K3XF (SybronEndo) made of R-phase NiTi alloy, and (iii) heat-treated K3 (K3H) which has received experimental heat treatment. The thermal treatment to K3H was performed for 30 minutes at a temperature of 500°C. K3H had a controlled memory wire (CM wire, DS Dental, Johnson City, TN, USA) like property that is extremely flexible. K3H can stay bent when it is precurved. They had #25 tip size with 0.06 taper and length of 21 mm identically. Before the test, every file was thoroughly inspected visually under OPMI dental microscope (Zeiss, Oberkochen, Germany) for detecting of defects.

### 2.2. Cyclic Fatigue Resistance Test

**Figure 1:**
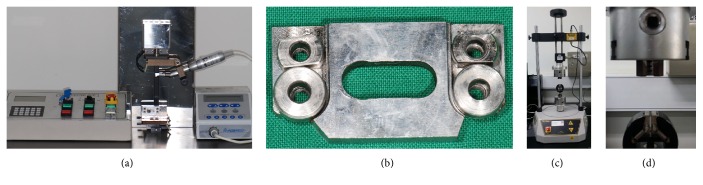
(a) Fatigue tester (Denbotix, Bucheon, Korea). (b) Artificial canal of fatigue tester; right part (curvature angle of 60°) was used in this study. (c) Torsion testing machine (Vortex-i, Macmesin Co., West Sussex, UK). (d) NiTi file engagement of (c) was magnified.

Ten files from each of three groups (*n* = 10) were used for this test. Cyclic fatigue resistance of the files was evaluated with a specially designed fatigue tester (Denbotix, Bucheon, Korea) (Figure 1(a)). The tester was composed of two components: an arm part and a metal artificial canal. The metal artificial canal (Figure 1(b)) had radius of 5 mm, intracanal diameter of 1.5 mm, a curvature angle of 60°, and a length of 8 mm at straight part. It was made of stainless steel. To confirm the fracture moment visually, the end of the artificial canal was open. The arm part was tightly holding the handpiece and able to move up and down. The up-and-down motion was set to oscillate 6 mm at a speed of 0.5 cycles per second to simulate clinical pecking motion. Each sample was mounted on a 20 : 1 reduction handpiece with an electric torque-control engine (Aseptico, Woodinville, WA, USA) and rotated clockwise at 300 rpm. The time to fracture was measured in seconds. The number of cycles to fracture (NCF) was obtained by multiplying the applied rpm (300 rpm) to the fracture time (minutes).

### 2.3. Torsional Resistance Test

Ten files from each of three groups (*n* = 10) were used for this test. Torsional resistance of the files was evaluated with a torsion testing machine (Vortex-i, Mecmesin Co., West Sussex, UK) (Figures 1(c) and 1(d)). The sample was mounted on the machine and tightly bound at the 5 mm point from the apical side. Until the fracture occurred, a clockwise rotation was applied at a speed of 2 rpm and torque and rotated angle were continuously recorded. From this test we obtained several mechanical properties of file, such as ultimate torsional strength (UTS), angle of rotation to fracture (ARF), and elastic modulus (EM). Since the elastic limit of a NiTi alloy is considered as much as 8% strain, we calculated the EM of each file within of 8% strain [9].

### 2.4. Scanning Electron Microscope (SEM) Analysis of Fractured Surfaces

After the file fractured, the fracture surfaces of handle side were examined under a SEM (Hitachi S-4700, Tokyo, Japan). And the unused files of each group were examined longitudinally under a SEM.

### 2.5. Statistical Analysis

The results were statistically analyzed by one-way ANOVA with SPSS statistical package version 22 (SPSS, Chicago, IL, USA). Tukey HSD and Games-Howell post hoc comparison were conducted at a significant level of 95%.

## 3. Results

### 3.1. Materials

As a result of experimental heat treatment, K3H showed enhanced flexibility. Furthermore, K3H could stay bent after being precurved. There features were similar to those of CM wire based NiTi files.

### 3.2. Cyclic Fatigue Resistance Tests

K3H showed much superior cyclic fatigue resistance (NCF) compared to K3 and K3XF. (*P* < 0.05) (Table 1). There was no significant difference in the cyclic fatigue resistance between K3 and K3XF (*P* > 0.05).

### 3.3. Torsional Resistance Test

**Figure 2:**
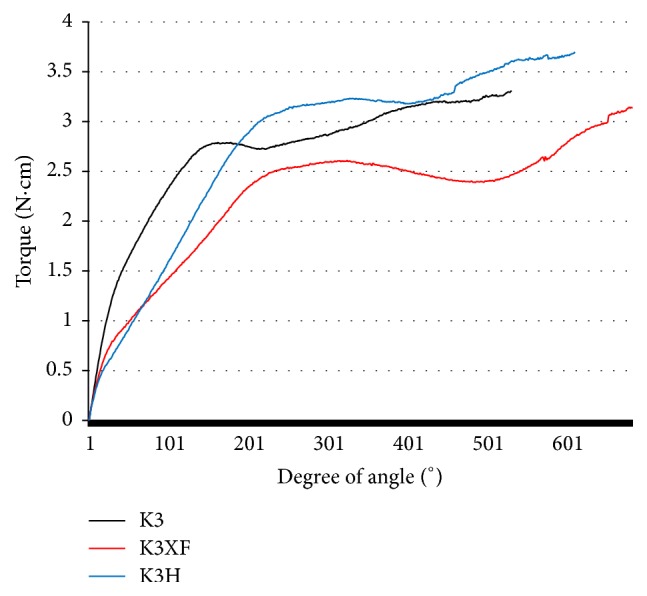
Mean torsional torque-rotated angle curves.

The mean torque-angle curves of tested files are shown in Figure 2. It shows EM, UTS, and ARF. The mean and standard deviation of the UTS and ARF for each instrument are presented in Table 1. The UTS was in the increasing order of K3XF, K3, and K3H, but there was no significant difference (*P* > 0.05). The ARF of K3XF is higher than that of K3 (*P* < 0.05). The difference in the ARF was not significant between K3XF and K3H, between K3H and K3 (*P* > 0.05). The EM of tested files is shown in Table 2. The EM of K3 was higher than that of K3XF and K3H (*P* < 0.05). There was no significant difference in the EM between K3XF and K3H (*P* > 0.05).

### 3.4. SEM Analysis

The SEM observations of the fracture surfaces of both tests showed typical appearance according to the type of tests.

**Figure 3:**
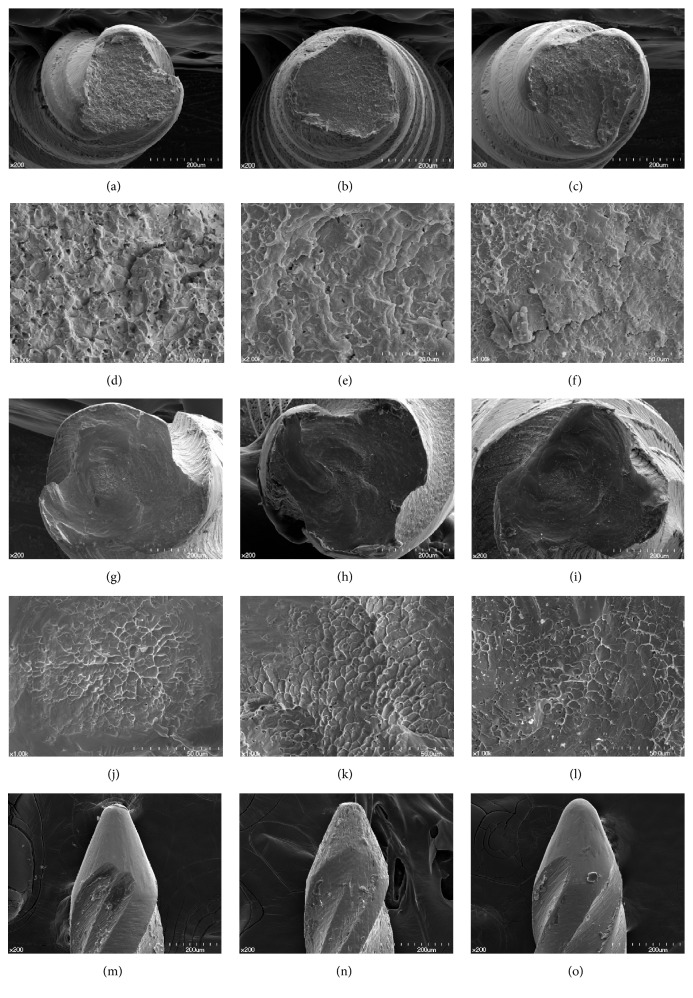
SEM images of fractured surfaces of files after cyclic fatigue fracture test (a–f) and torsional fracture test (g–l). SEM images of lateral surface of unused files (m–o). (a, d, g, j, m) K3, (b, e, h, k, n) K3XF, and (c, f, i, l, o) K3H.

Cyclic fatigue fractured surfaces showed one or more crack origins and crack growth from each crack origin. Multiple striations, a characteristic of fatigue failure, were observed in high magnification. Each striation was marking the momentary position of the crack propagation during compressive stress. And this crack growth manifested as microscopic dimples of irregular shape and size (Figures 3(a)–3(f)).

In the case of torsional fracture, concentric circular markings appeared at the periphery with a “fibrous” appearance in the center. In high magnification, the skewed dimples, a characteristic of ductile fracture, were observed at the fibrous region. These typical fracture patterns were shown in all samples observed, and there was no notable difference between the three instruments (Figures 3(g)–3(l)).

When observing the unused sample of each file longitudinally, K3 and K3H showed almost the same appearance. However, K3XF showed many shallow hollows on the surface (Figures 3(m)–3(o)).

## 4. Discussion

There have been many attempts to improve the fracture resistance of NiTi files. Recently, many studies have introduced the thermal treatments of NiTi alloy [10, 11]. This opened up new generations of NiTi file. NiTi file manufactured with M-wire (Dentsply Maillefer, Ballaigues, Switzerland) was introduced in 2007. It is manufactured through a series of thermal treatments on NiTi alloy blanks. It showed improved flexibility compared to conventional NiTi alloy [12]. M-wire files include Dentsply's WaveOne. R-phase NiTi alloy (SybronEndo) was introduced in 2008. R-phase is a phase present in a very narrow temperature range between austenite and martensite, with a rhombohedral structure. R-phase NiTi files include SybronEndo's TF and K3XF. CM wire is a novel NiTi alloy introduced in 2010. It is manufactured through a special thermal treatment and controls the memory of material, making the alloy extremely flexible, compared to other NiTi files [8]. HyFlex (Coltene/Whaledent, Inc, Cuyahoga Falls, OH, USA) and Typhoon (Clinician's Choice Dental Products, New Milford, CT, USA) are CM wire NiTi files.

K3XF was manufactured using R-phase technology while having basic features of original K3. Thus, the manufacturers claim that K3XF has more flexibility and resistance to cyclic fatigue. And K3H, made from experimental heat treatment to the original K3, showed the characteristic similar to CM wire which maintains its shape when bent without rebounding. Because all the three files, used in this study, have the same design, the geometric factor had no influence in the outcomes.

For the cyclic fatigue test, the situation similar to a curved root canal should be reproduced. There are four methods to reproduce a curved root canal experimentally: (i) curved metal tube, (ii) grooved block-and-rod assembly, (iii) inclined plane, and (iv) three-point bending device [13]. In this study, we used block-and-rod assembly made of stainless steel as an artificial canal and reproduced a situation similar to clinical procedure with apical pecking motion of the handpiece.

In the literature, it was reported that the thermal treatment to the NiTi alloy increased flexibility by changing the composition of phases [14]. In this study, the EM of K3 was significantly higher than that of K3XF and K3H. In other words, K3XF and K3H were more flexible than K3. The resistance to cyclic fatigue is related to the flexibility of a file. The cyclic fatigue resistance of K3H was significantly higher than the other two groups. On the other hand, K3XF showed higher NCF than K3, but the difference was not significant. When compared to other studies that reported K3XF has a greater cyclic fatigue resistance than K3, there was a difference in this study [5, 15, 16].

While manufacturing NiTi files, small scratches and grooves are generated [4]. The initiation of fatigue cracks usually occurs at the surface defect of files and the crack progresses along the machining grooves [17]. These defects are inevitable since both K3 and K3XF are manufactured through milling process. According to SEM analysis, small scratches were observed on the surfaces of all files. In addition, a number of shallow hollows were observed on the surface of K3XF. In spite of having higher flexibility, K3XF did not show higher resistance to the cyclic fatigue. It is assumed that these hollows might affect the failure. On the other hand, K3H, not different from K3 in the surface structure, showed higher flexibility and cyclic fatigue resistance. This is consistent with previous study which claimed that CM wire was more resistant to fatigue failure than conventional NiTi alloy [18].

Torsional fracture occurs when a file tip is bound in the canal, but the motor continues to rotate. When the elastic limit on the file is exceeded, plastic deformation occurs and finally the file fractures. Two conditions are needed for torsional fracture: (i) the stress exceeding the UTS and (ii) the deformation exceeding the ARF. Therefore, to improve torsional resistance, UTS and plasticity of a file should be improved.

UTS is the ability to resist torsional fracture. In this study, there was no significant difference among all three groups in UTS. During the thermal treatment process, a file undergoes softening and annealing, which makes it more flexible. Generally, flexible files have been assumed to be less resistant to torsional stress. However, there was no significant decrease in UTS after thermal treatment in this study.

Plasticity is defined as the ability to be plastically deformed under stress without failure, and it is represented by ARF in this study. It is considered that the increase of plasticity of thermally treated file is due to the increase of proportions of R-phase and martensite. R-phase has the lowest shear modulus among the three phases [19]. And martensite is more likely to deform compared to austenite since martensite has twinning process, an internal movement of lattices without breaking atomic bonds by absorbing stress [20]. In this study, K3XF was more plastic than K3 significantly. And K3H was also more plastic than K3, but the difference was not significant.

Plasticity is considered more important factor than UTS in torsional fracture [21]. It is because that plastic deformation can act as a safety factor before torsional fracture [22]. This plastic deformation can be seen visually when withdrawing a file from a root canal, and it can give a warning that torsional fracture is imminent. In this respect, K3XF is regarded safer than K3 in torsional fracture.

## 5. Conclusions

Compared to K3 which is made of conventional NiTi alloy, K3XF (made of R-phase NiTi alloy) and K3H (heat treated experimentally to the original K3) showed increased flexibility without decrease of the other mechanical properties such as UTS and plasticity. Particularly, K3H showed a far superior cyclic fatigue resistance to other groups.

## Figures and Tables

**Table 1:** Cyclic fatigue resistance and torsional resistance (mean ± standard deviation).

	Cyclic fatigue resistance	Torsional resistance	
	NCF	UTS (Ncm)	ARF (°)
K3	1683.0 ± 349.4^a^	3.31 ± 0.55^a^	526.4 ± 110.7^a^
K3XF	1995.0 ± 295.5^a^	3.14 ± 0.42^a^	678.0 ± 96.5^b^
K3H	17760.5 ± 5199.4^b^	3.70 ± 0.73^a^	605.1 ± 133.0^a,b^

Different superscript letters in the same column indicate a statistically significant difference between groups (*P* < 0.05).

NCF: number of cycles to fracture; UTS: ultimate torsional strength; ARF: angle of rotation to fracture.

**Table 2:** Elastic modulus of NiTi files (mean ± standard deviation).

	Elastic modulus (Ncm/*θ*)
K3	0.43 ± 0.08^a^
K3XF	0.27 ± 0.06^b^
K3H	0.22 ± 0.05^b^

Different superscript letters indicate a statistically significant difference between groups (*P* < 0.05).
